# One–Pot Phosphate-Mediated Synthesis of Novel 1,3,5-Trisubstituted Pyridinium Salts: A New Family of *S. aureus* Inhibitors

**DOI:** 10.3390/molecules22040626

**Published:** 2017-04-12

**Authors:** Thomas Pesnot, Markus C. Gershater, Martin Edwards, John M. Ward, Helen C. Hailes

**Affiliations:** 1 Department of Chemistry, University College London, Christopher Ingold Laboratories, 20 Gordon Street, London WC1H 0AJ, UK; t.pesnot@redxpharma.com; 2 The Advanced Centre for Biochemical Engineering, Department of Biochemical Engineering, University College London, Bernard Katz Building, Gower Street, London WC1E 6BT, UK; m.gershater@synthace.com (M.C.G.); j.ward@ucl.ac.uk (M.E.); j.ward@ucl.ac.uk (J.M.W.)

**Keywords:** heteroaromatic synthesis, Chichibabin reaction, synthesis in water, pyridinium salts, antibacterial activity

## Abstract

Polysubstituted pyridinium salts are valuable pharmacophores found in many biologically active molecules. Their synthesis typically involves the use of multistep procedures or harsh reaction conditions. Here, we report water-based phosphate mediated reaction conditions that promote the condensation of arylacetaldehydes with amines to give 1,3,5-pyridinium salts. The reaction, carried out at pH 6, provides conditions suitable for the use of less stable aldehydes and amines in this Chichibabin pyridine condensation. The evaluation of selected 1,3,5-trisubstituted pyridinium salts highlighted that they can inhibit the growth of *S. aureus* in the low μg/mL range. The synthetic accessibility of these compounds and preliminary growth inhibition data may pave the way towards the discovery of new anti-bacterials based on the 1,3,5-trisubstituted pyridinium scaffold.

## 1. Introduction

**Figure 1:**
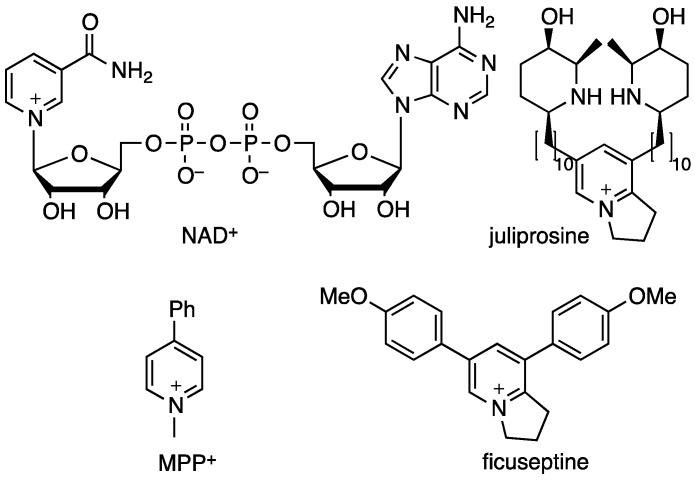
Examples of biologically active pyridinium salts.

Polysubstituted pyridine and pyridinium salts such as the coenzymes NAD^+^ and NADP^+^ [1] are involved in many essential biochemical processes. They are also part of other biologically active natural products such as juliprosine [2], the antibacterial alkaloid ficuseptine [3,4,5], and the neurotoxic metabolite 1-methyl-4-phenylpyridinium (MPP^+^) [6,7] (Figure 1). In addition, synthetic pyridinium species have been developed to facilitate gene delivery [8], or act as platelet activation antagonists [9], and benzylidenehydrazinyl pyridiniums have been investigated as antimicrobial agents [10]. The synthesis of polysubstituted pyridiniums however typically involves multistep procedures, or harsh reaction conditions, and the development of rapid and mild methodologies for the facile preparation of new polysubstituted pyridinium analogues is sought after.

The Chichibabin reaction for the synthesis of polysubstituted pyridines was first reported nearly 100 years ago [11]. The reaction involves the condensation of aldehydes with an amine to yield pyridiniums in a single synthetic step. Initially, the reaction required elevated temperatures and pressure, or acid catalysis [12,13,14,15,16,17], but the intrinsic instability of aldehydes under these forcing conditions often resulted in product yields that were at best mediocre.

**Scheme 1:**
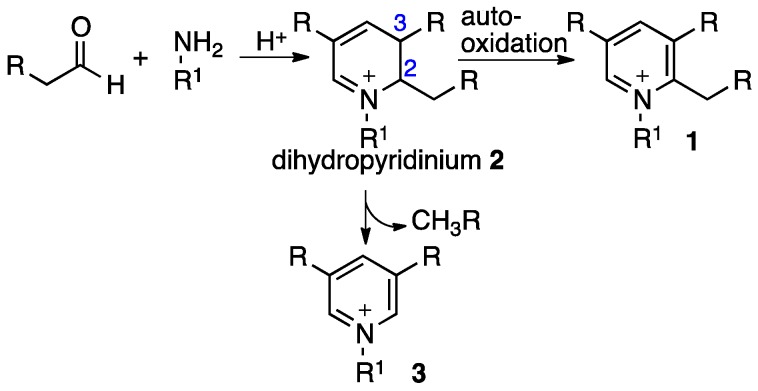
Three products **1**–**3** isolated from the acid-mediated Chichibabin reaction.

Typically, three products can be isolated from the acid-mediated condensation of amines with acetaldehydes in the Chichibabin reaction (Scheme 1); a 1,2,3,5-tetrasubstituted pyridinium **1** (often the major product), which is formed via the auto-oxidation of the product 1,2,3,5-dihydropyridinium **2**, and 1,3,5-trisubstituted pyridinium salts **3** (a minor product often referred to as the ‘abnormal’ Chichibabin product).

Triflate salts of lanthanides have been used in room temperature reactions to promote the Chichibabin condensation reaction between amines and acetaldehydes, giving **1**, **2**, or **3** in varying amounts [18]. More recently, the condensation of benzylamine and arylacetaldehydes in a 50 mol % solution of ytterbium triflate in water provided the first report of a selective synthesis of pyridinium salts **3** [19,20,21]. However, the use of such rare-earth Lewis acid catalysts raises sustainability and cost issues. Glacial acetic acid [21] was also reported to promote the condensation of phenethylamine and phenylacetaldehyde to the pyridinium **3**, but such harsh reaction conditions are unsuitable for intrinsically unstable substrates such as arylacetaldehydes.

In recent studies we have reported that phosphate can catalyse the Pictet-Spengler condensation reaction of aldehydes with amines to give tetrahydroisoquinoline alkaloids (THIAs) [22,23,24]. Phosphate was proposed to catalyse the reaction by favouring formation of the imine intermediate, and participating in the phenolic deprotonation. Imine formation and activation are also essential to the Chichibabin pyridine synthesis, and it was therefore anticipated that phosphate might promote the synthesis of polysubstituted pyridiniums in water. Here we report the use of mild aqueous phosphate-based reaction conditions to provide access to 1,3,5-pyridinium salts **3** and outline the versatility of the reaction. The resulting compounds were evaluated as new bacterial growth inhibitors against *S. aureus*.

## 2. Results and Discussion

**Scheme 2:**
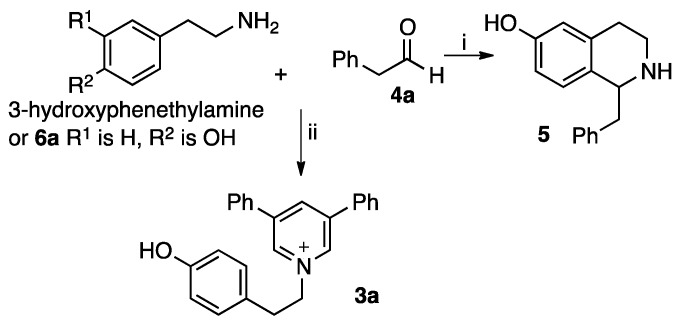
Products **3a** and **5** resulting from the condensation of **4a** with either tyramine **6a** or 3-hydroxyphenethylamine, respectively. *Reagents and conditions*: (i) 3-hydroxyphenethylamine, 0.1 M KPi, pH 6, 60 °C, 12 h; (ii) **6a**, 0.1 M KPi, pH 6, 60 °C, 12 h.

We have recently reported a phosphate mediated biomimetic Pictet-Spengler synthesis of THIAs [22]. The mild reaction conditions allowed even the least stable arylacetaldehydes to generate THIAs in good yields [22,23,24]. This biomimetic Pictet-Spengler condensation reaction is selective towards the amine component, requiring a phenethylamine substrate that is *meta*-substituted with a strong electron-donating group (e.g., a hydroxyl group) such as in 3-hydroxyphenethylamine. The condensation of this phenethylamine for example with an aldehyde such as phenylacetaldehyde **4a** in potassium phosphate (KPi) buffer gave the THIA **5** (Scheme 2). Chichibabin and Pictet-Spengler condensations both involve amine and aldehyde components, imine formation and activation, and therefore by analogy with the Pictet-Spengler reaction it was anticipated that the Chichibabin reaction could be promoted by phosphates. This hypothesis was investigated by reacting phenylacetaldehyde **4a** with tyramine (4-hydroxyphenethylamine) **6a** (unreactive under biomimetic Pictet-Spengler conditions) in a 1.2:1 ratio (as reported for the synthesis of THIAs) in KPi buffer (pH 6), at 60 °C for 12 h. As expected no THIA was formed, but several compounds were detected in trace quantities, with the main product of the reaction (5% yield) being the ‘abnormal’ Chichibabin pyridinium salt **3a** (Scheme 2).

In order to optimise reaction conditions to favour formation of the pyridinium product **3a**, the ratio of **4a** to **6a** was increased from 1.2:1 to 5:1 to account for the stoichiometry of the reaction, and a range of buffers were screened at 0.1 M (pH 6) as media for the reaction (Table 1).

The four phosphate-based buffers tested (Table 1, entries 1–4) catalysed the production of pyridinium salt **3a**, whereas other buffers or water alone (Table 1, entries 5–8) did not significantly promote the reaction. These results suggested that phosphates promote not only the synthesis of THIAs, but also that of 1,3,5-trisubstituted pyridinium salts **3**. All four phosphates, glucose-1-phosphate (Glc-1-P), inorganic pyrophosphate (PPi), uridine 5′-phosphate (UMP) and inorganic phosphate (Pi) are naturally abundant and essential to cell survival. They are involved in buffering cellular pH, for storing genetic information (UMP and other nucleotide building blocks for DNA and RNA) and transferring biochemical information (PPi is a by-product of the hydrolysis of ATP mediated by kinases). Hence, the synthesis of 1,3,5-trisubstituted pyridiniums, as well as THIAs, is likely to proceed at low levels under mild conditions in vivo. This could explain the occurrence of plant natural products such as the haouamines [19]. The in vivo condensation of aldehydes such as allysine with itself or with lysine has also been reported: the resulting polyfunctional pyridinium salt cross-links in elastin is believed to be an age-related intermolecular cross-linking process [25,26].

The reaction conditions were further improved by increasing the KPi buffer concentration to 0.25 M and elevating the temperature to 100 °C. The reaction pH was however kept at 6 since more acidic conditions resulted in slower reaction rates and more alkaline conditions favoured the polymerisation of phenylacetaldehyde. Finally, the solubility of both amine and aldehyde substrates was enhanced by using a 1:1 mixture of KPi buffer with methanol. The combination of these new conditions was rewarded with the production of the **3a** in 70% isolated yield. Aldehyde **4a** was then reacted with a range of (hetero)arylethylamines **6b**–**6g**, a benzylamine **6h**, aliphatic amines **6i**–**6l**, and aromatic and heteroaromatic amines **6m**, **6n** to form the corresponding 1,3,5-trisubstituted pyridinium salts **6b**–**6l** in 13%–72% isolated yields (Table 2). Functionalities including hydroxyls, halogens and carboxylates were well tolerated. The lack of reactivity observed with aromatic amines such as aniline **6m** or 2-aminobenzimidazole **6n** may originate from their poor nucleophilicity as well as steric hindrance.

In contrast to the amines, the reaction was highly selective towards aldehyde substrates (Table 3). While tyramine **6a** reacted with arylacetaldehydes and (hetero)arylacetaldehydes, no reaction was observed with aliphatic aldehydes with the exception of 3-methylbutyraldehyde **4r**: reaction of **6a** with **4r** produced the pyridinium **3r** in a low yield of 5% and also the pyridinium **1r** (10% yield). The formation of **1r** suggested that the phosphate-mediated reaction was analogous to a Chichibabin type condensation where the aldehyde directed the reaction towards either the Chichibabin product **1** via an oxidation reaction or 1,3,5-trisubstituted pyridiniums **3** via an elimination step (Scheme 1).

These results are consistent with the recent work published by Baran et al. on the Yb(OTf)_3_-mediated Chichibabin synthesis of pyridinium salts [19,20]. They suggested that the lanthanide salt acts as a Lewis acid catalyst and promotes the synthesis of **3**, with benzylic group removal at C-2 via oxidation and formation of benzaldehyde. Alternatively, Poupon et al. suggested that toluene may be generated upon pyridinium aromatisation [21]. In this study, we have demonstrated that the synthesis of **3** can also be promoted by phosphates in good isolated yields. Phosphate acid/base catalysis can promote keto-enol equilibria, proton transfers, and eliminations, which typically occur during the Chichibabin pyridine synthesis. Here, the excellent nucleophilic and leaving group abilities of phosphates may further facilitate the reaction. This concept is reminiscent of investigations by Sutherland and co-workers on the prebiotic synthesis of nucleobases in which phosphate is described as a general acid/base catalyst as well as a nucleophile activating nitriles for subsequent condensation reactions [27]. Here, in the final elimination step producing **3**, loss of proton 3-H can be assisted by phosphate, and the substituent at C-2 then leaves. If the substituent at C-2 is a good leaving group (toluene in the case of arylacetaldehydes), 1,3,5-trisubstituted pyridiniums **3** will predominantly be formed (Scheme 1). Otherwise, the 1,2,3,5-tetrasubstituted intermediate will slowly oxidise to generate the corresponding 1,2,3,5-tetrasubstituted pyridinium Chichibabin salt **1**.

There is a growing need for new antibiotics and over the last 30 years the number of new antibiotics entering the clinic has decreased significantly. During that time there has been a constant rise in the resistance of pathogenic bacteria to the antibiotics in use. One of the major pathogens in hospitals and increasingly in the community is *Staphylococcus aureus*. This bacterium has acquired resistance over the last 25 years to methicillin and in the last 15 years to vancomycin. In the light of this need for new antibiotics to treat *S. aureus* infections, and the anti-microbial activities reported for ficuseptine [3], selected 1,3,5-pyridinium compounds were tested for their ability to inhibit the growth of *S. aureus.* Agar plate diffusion assays and liquid minimal inhibitory concentration (MIC) tests were carried out to assess the potency of the compounds.

**Figure 2:**
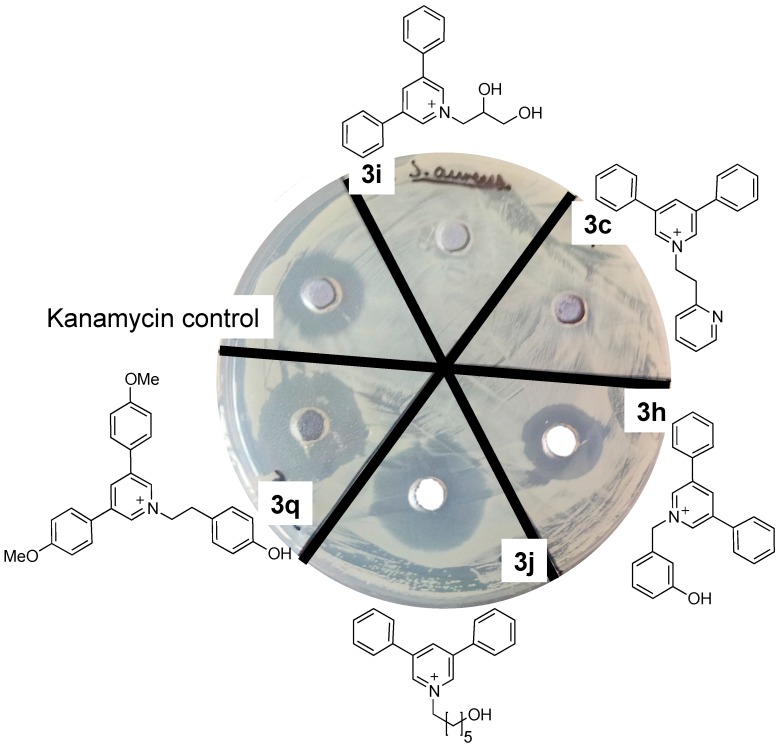
Examples of the agar plate diffusion assays against *S. aureus* with selected pyridiniums **3** (at 400 μg/mL) and the kanamycin control (at 100 μg/mL).

Some examples of the agar plate diffusion assays against a kanamycin control are shown in Figure 2 and the MIC data in Table 4. The data demonstrated the potency of some of the compounds compared to the antibiotic kanamycin.

**Figure 3:**
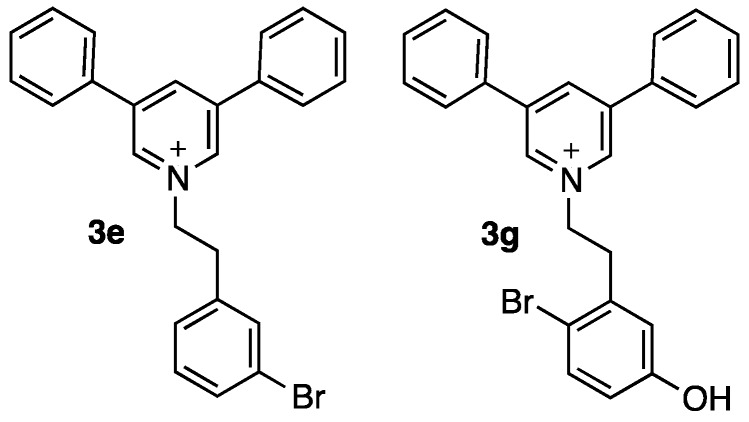
Structures of **3e** and **3g**.

From the compounds tested it was notable that the two most potent anti-bacterials against *S. aureus* were compounds **3e** and **3g** (Figure 3). Both had an aromatic ring at C-3 and C-5 of the pyridinium ring, indeed for **3r** where an isopropyl group was present, no growth inhibitory properties were noted. In addition, **3g** had a 5-bromo-3-hydroxyphenyl group attached via an ethyl spacer to N-1, whereas in **3e** a 3-bromophenyl group was present. Overall this data suggests that aromatic substituents at positions C-3 and C-5 are preferred, and that substitution of the phenyl rings is tolerated, as demonstrated by **3o** and **3q**. Also, that substitution of the N-1 phenethyl group by a halogen leads to good inhibitory growth properties. However, it is not essential for an aromatic group to be present at N-1, for example as with **3j**, for inhibitory growth effects to be conferred.

These results point to compounds that can now be made to explore the structure activity relationship of the various substituents that can be placed on the C-1, C-3, and C-5 positions of the pyridine ring. The activity against other bacteria will be reported elsewhere.

## 3. Experimental Section

### 3.1. General Information and Methods

#### 3.1.1. Chemistry

All reagents were obtained from commercial sources and used as received unless otherwise stated. TLC was performed on Kieselgel 60 F_254_ precoated plastic plates and compounds visualised by exposure to UV light, potassium permanganate, phosphomolybdic acid (PMA) or ninhydrin. Flash column chromatography was carried out using silica gel (particule size 40–63 μm). Preparative HPLC were performed on a Prostar instrument (Varian Inc., Middelburg, The Netherlands) equipped with an autosampler, a UV-visible detector and a DiscoveryBIO wide Pore C18-10 Supelco column (25 × 2.12 cm). Elutions were monitored at 280 nm and carried out using a gradient of 5% to 90% acetonitrile against water (+0.1% trifluoroacetic acid). NMR: ^1^H- and ^13^C-NMR spectra were recorded at 298 K at the field indicated using Avance 500 and Avance III 600 spectrometers. (Bruker, (UK) Ltd., Coventry, UK). Coupling constants (*J*) are measured in Hertz (Hz) and multiplicities for ^1^H-NMR couplings are shown as s (singlet), d (doublet), t (triplet), hept (heptet) and m (multiplet). Chemical shifts (in ppm) are given relative to tetramethylsilane and referenced to residual protonated solvent. Infrared spectra were recorded on a Spectrum 100 FTIR spectrometer (Perkin Elmer, Shelton, CT, USA). Mass spectrometry analyses were performed at the UCL Chemistry Mass Spectrometry Facility using a Finnigan MAT 900 XP and MicroMass Quattro LC mass spectrometer (Waters UK, Elstree, UK). TFA refers to the CF_3_CO_2_^−^ salt and NMR signals are not recorded for TFA in the ^13^C-NMR data.

#### 3.1.2. Biological Assays

##### Plate Zone Assays

*Staphylococcus aureus* was grown overnight in LB broth (Oxoid Ltd., Basingstoke, UK) and 100 μL was spread onto the surface of LB agar plates using a sterile spreader. A sterile glass pipette with a diameter of 6 mm was used to punch out wells and the agar removed from the wells. 50 μL of compound, at a concentration of 400 μg/mL in RO water, or a control solution (positive control kanamycin at 100 μg/mL; negative control 1:12.5 DMSO/water) was pipetted into each well. The LB agar plates were then left to incubate at 37 °C for 18–24 h. The zone diameters were measured at 18 h.

##### Minimimal Inhibitory Concentration (MIC)

Fifty mL of LB broth was inoculated with a 1 mL overnight culture of *Staphylococcus aureus*, then 450 μL of the inoculated broth was pipetted into each well of a 96 deep square well plate. 50 μL of compound was pipetted into each well; the compound was taken from the 256 μg/mL to 2 μg/mL dilution series. A 5 mg/mL stock concentration of the test compounds was diluted with Reverse Osmosis (RO) water in clean, sterile 1.5 mL Eppendorf Micro-centrifuge tubes to a starting concentration of 256 μg/mL. A 2-fold dilution series was made using RO water down to a concentration 2 μg/mL. There were 8 different dilutions in each series ranging from 256 μg/mL to 2 μg/mL. After growth of the plate at 37 °C for 18–24 h with shaking, the wells with no growth were recorded as the MIC.

### 3.2. Procedure A for Synthesis of the 1,3,5-Substituted Pyridinium Salts

Amine (1 equiv.) and aldehyde (5 equiv.) were dissolved in a 1:1 mixture of methanol/phosphate buffer (10 mL, 0.25 M solution at pH 6). The resulting solution was stirred at 100 °C for 12 h. The crude mixture was cooled to r.t. and purified by preparative HPLC (Gradient 1). Fractions containing the desired product were combined, concentrated under vacuum and co-evaporated with methanol (3 × 20 mL).

*1-(4-Hydroxyphenethyl)-3,5-diphenylpyridinium***·***TFA* (**3a·TFA**). Compound **3a** was prepared according to procedure A from tyramine**·**HCl (87 mg, 0.50 mmol) and phenylacetaldehyde (300 mg, 2.50 mmol). The crude product was purified by preparative HPLC (retention time 15.5 min) to give **3a·TFA** as a pale yellow oil (162 mg, 70%). ν_max_ (neat)/cm^−1^ 3064, 1671, 1613, 1596, 1517; ^1^H-NMR (500 MHz; CD_3_OD) δ 3.29–3.32 (2H, m, C*H*_2_CH_2_N^+^), 4.95 (2H, t, *J* = 6.7 Hz, C*H*_2_N^+^), 6.74 (2H, d, *J* = 8.5 Hz, 3′′-H, 5′′-H), 6.98 (2H, d, *J* = 8.5 Hz, 2′′-H, 6′′-H), 7.56–7.60 (6H, m, Ph), 7.72–7.76 (4H, m, Ph), 8.91 (2H, d, *J* = 1.7 Hz, 2-H, 6-H), 8.95 (1H, t, *J* = 1.7 Hz, 4-H); ^13^C-NMR (125 MHz; CD_3_OD) δ 37.5, 64.6, 116.7, 127.2, 128.5, 130.6, 131.1, 131.3, 134.5, 141.4, 141.7, 142.5, 158.1; *m*/*z* [HRMS ES+] found [M − TFA]^+^ 352.1700. C_25_H_22_NO^+^ requires 352.1701.

*1-Phenethyl-3,5-diphenylpyridinium***·***TFA* (**3b**·**TFA**). Compound **3b** was prepared according to procedure A from phenethylamine (61 mg, 0.50 mmol) and phenylacetaldehyde (300 mg, 2.50 mmol). The crude product was purified by preparative HPLC (retention time 16.5 min) to give **3b**·**TFA** as a pale yellow oil (121 mg, 54%). ν_max_ (neat)/cm^−1^ 3064, 1683, 1597, 1482; ^1^H-NMR (500 MHz; CD_3_OD) δ 3.50 (2H, t, *J* = 6.9 Hz, C*H*_2_CH_2_N^+^), 5.10 (2H, t, *J* = 6.9 Hz, C*H*_2_N^+^), 7.28 (2H, d, *J* = 6.8 Hz, 2′′-H, 6′′-H), 7.33–7.41 (3H, m, Ph), 7.64–7.66 (6H, m, Ph), 7.80–7.83 (4H, m, Ph), 9.05 (1H, t, *J* = 1.6 Hz, 4-H), 9.07 (2H, d, *J* = 1.6 Hz, 2-H, 6-H); ^13^C-NMR (125 MHz; CD_3_OD) δ 38.2, 64.2, 117.9, 128.5, 130.0, 130.5, 131.4, 134.5, 136.9, 141.6, 141.7, 142.6; *m*/*z* [HRMS ES+] found [M − TFA]^+^ 336.1750. C_25_H_22_N^+^ requires 336.1752.

*3,5-Diphenyl-1-[2-(pyridin-2-yl)ethyl]pyridinium***·***TFA* (**3c·TFA**). Compound **3c** was prepared according to procedure A from 2-(2-aminoethyl)pyridine (35 mg, 0.29 mmol) and phenylacetaldehyde (170 mg, 1.4 mmol). The crude product was purified by preparative HPLC (retention time 13.5 min) to give **3c·TFA** as a pale yellow oil (68 mg, 52%). ν_max_ (neat)/cm^−1^ 3077, 1635, 1598, 1483; ^1^H-NMR (500 MHz; CD_3_OD) δ 3.85 (2H, t, *J* = 7.4 Hz, C*H*_2_CH_2_N^+^), 5.21 (2H, t, *J* = 7.4 Hz, C*H*_2_N^+^), 7.57–7.64 (6H, m, Ph), 7.73 (1H, dd, *J* = 7.6 and 4.9 Hz, 5′′-H), 7.83 (1H, d, *J* = 7.6 Hz, 3′′-H), 7.87–7.89 (4H, m, Ph), 8.27 (1H, t, *J* = 7.6 Hz, 4′′-H), 8.68 (1H, d, *J* = 4.9 Hz, 6′′-H), 9.04 (1H, t, *J* = 1.5 Hz, 4-H), 9.27 (2H, d, *J* = 1.5 Hz, 2-H, 6-H); ^13^C-NMR (125 MHz; CD_3_OD) δ 37.3, 61.6, 126.0, 127.8, 128.8, 129.5, 130.8, 131.6, 134.7, 142.2, 142.3, 143.2, 144.4, 146.6, 154.5; *m*/*z* [HRMS ES+] found [M − TFA]^+^ 337.1708. C_24_H_21_N_2_^+^ requires 337.1705.

*3,5-Diphenyl-1-[2-(thiophen-2-yl)ethyl]pyridinium***·***TFA* (**3d**·**TFA**). Compound **3d** was prepared according to procedure A from thiophene-2-ethylamine (36 mg, 0.28 mmol) and phenylacetaldehyde (170 mg, 1.4 mmol). The crude product was purified by preparative HPLC (retention time 16.2 min) to give **3d**·**TFA** as a pale yellow oil (48 mg, 38%). ν_max_ (neat)/cm^−1^ 3065, 2940, 1680, 1597, 1483; ^1^H-NMR (500 MHz; CD_3_OD) δ 3.67 (2H, t, *J* = 6.6 Hz, C*H*_2_CH_2_N^+^), 5.02 (2H, t, *J* = 6.6 Hz, C*H*_2_N^+^), 6.87 (1H, m, 3′′-H), 6.96 (1H, dd, *J* = 5.1 and 3.5 Hz, 4′′-H), 7.33 (1H, dd, *J* = 5.1 and 1.1 Hz, 5′′-H), 7.56–7.62 (6H, m, Ph), 7.77–7.79 (4H, m, Ph), 8.98 (1H, t, *J* = 1.7 Hz, 4-H), 9.04 (2H, d, *J* = 1.7 Hz, 2-H, 6-H); ^13^C-NMR (125 MHz; CD_3_OD) δ 32.2, 64.4, 126.7, 128.5, 128.7, 128.8, 130.8, 131.6, 134.7, 138.5, 141.9, 142.0, 142.9; *m*/*z* [HRMS ES+] found [M − TFA]^+^ 342.1302. C_23_H_20_NS^+^ requires 342.1316.

*1-(2-[3-Bromophenethyl])-3,5-diphenylpyridinium***·***TFA* (**3e·TFA**). Compound **2e** was prepared according to procedure A from 3-bromophenethylamine (100 mg, 0.50 mmol) and phenylacetaldehyde (300 mg, 2.5 mmol). The crude product was purified by preparative HPLC (retention time 17.2 min) to give **3e·TFA** as a pale yellow oil (131 mg, 50%). ν_max_ (neat)/cm^−1^ 3086, 2979, 1685, 1601, 1488; ^1^H-NMR (500 MHz; CD_3_OD) δ 3.42 (2H, t, *J* = 7.2 Hz, C*H*_2_CH_2_N^+^), 5.00 (2H, t, *J* = 7.2 Hz, C*H*_2_N^+^), 7.21–7.27 (2H, m, 2′′-H, 5′′-H), 7.42–7.47 (2H, m, 4′′-H, 6′′-H), 7.57–7.61 (6H, m, Ph), 7.79 (4H, d, *J* = 6.3 Hz, 2 × 2′-H, 6′-H), 8.97 (1H, t, *J* = 1.7 Hz, 4-H), 9.09 (2H, d, *J* = 1.7 Hz, 2-H, 6-H); ^13^C-NMR (125 MHz; CD_3_OD) δ 37.8, 63.9, 124.0, 128.8, 129.1, 130.8, 131.6, 131.8, 131.9, 133.3, 134.7, 139.8, 141.9, 142.0, 143.0; *m*/*z* [HRMS ES+] found [M − TFA]^+^ 414.0840. C_25_H_21_^79^BrN^+^ requires 414.0857.

*1-[2-(3-Nitrophenethyl)]-3,5-diphenylpyridinium***·***TFA* (**3f·TFA**). Compound **3f** was prepared according to procedure A from 3-nitrophenethylamine (17 mg, 0.10 mmol) and phenylacetaldehyde (60 mg, 0.5 mmol). The crude product was purified by preparative HPLC (retention time 16.0 min) to give **3f·TFA** as a pale yellow oil (22 mg, 45%). ν_max_ (neat)/cm^−1^ 3070, 1655, 1598, 1561; ^1^H-NMR (500 MHz; CD_3_OD) δ 3.59 (2H, t, *J* = 7.4 Hz, C*H*_2_CH_2_N^+^), 5.04 (2H, t, *J* = 7.4 Hz, C*H*_2_N^+^), 7.59–7.64 (7H, m, 5′′-H and Ph), 7.71 (1H, d, *J* = 7.6 Hz, 6′′-H), 7.83 (4H, dd, *J* = 7.8 and 1.7 Hz, 2 × 2′-H, 6′-H), 8.16 (1H, d, *J* = 8.2 Hz, 4′′-H), 8.20 (1H, s, 2′′-H), 9.04 (1H, t, *J* = 1.6 Hz, 4-H), 9.21 (2H, d, *J* = 1.6 Hz, 2-H, 6-H); ^13^C-NMR (125 MHz; CD_3_OD) δ 37.7, 63.6, 123.6, 125.1, 128.8, 130.8, 131.4, 131.6, 134.7, 136.6, 139.5, 142.0, 142.1, 143.1, 149.5; *m*/*z* [HRMS ES+] found [M − TFA]^+^ 381.1604. C_25_H_21_N_2_O_2_^+^ requires 381.1603.

*3-(2-Aminoethyl)-4-bromophenol* (**6g**). The reaction was carried out under anhydrous conditions. To a solution of 2-(2-bromo-5-methoxyphenyl)ethylamine [29] (120 mg, 0.52 mmol) in dichloromethane (20 mL) at −78 °C, boron tribromide (1.3 mL, 1.3 mmol; 1 M solution in hexane) was added. The mixture was warmed to r.t. and stirred for 20 h, then cooled to −78 °C and water (50 mL) added dropwise. The aqueous layer was extracted with dichloromethane (3 × 50 mL), filtered and concentrated under reduced pressure to give **6g** [30] as a colourless oil (110 mg, 97%). ^1^H-NMR (600 MHz; D_2_O) δ 3.09 (2H, t, *J* = 7.5 Hz, C*H*_2_CH_2_N), 3.29 (2H, t, *J* = 7.5 Hz, C*H*_2_N), 6.77 (1H, dd, *J* = 8.7 and 3.0 Hz, 6-H), 6.90 (1H, d, *J* = 3.0 Hz, 2-H), 7.52 (1H, d, *J* = 8.7 Hz, 5-H); ^13^C-NMR (150 MHz; D_2_O) δ 33.9, 39.7, 114.4, 117.1, 118.7, 134.6, 137.7, 156.1; *m*/*z* [HRMS ES+] found [MH]^+^ 216.0030. C_8_H_11_^79^BrNO requires 216.0024.

*1-(2-Bromo-5-hydroxyphenethyl)-3,5-diphenylpyridinium***·***TFA* (**3g·TFA**). Compound **6g** (50 mg, 0.23 mmol) and phenylacetaldehyde (33 mg, 0.28 mmol) in 10 mL of a 1:1 mixture of acetonitrile/phosphate buffer (0.1 M solution at pH 6) were stirred at 60 °C for 12 h. The crude product was purified by preparative HPLC (retention time 16.5 min) and fractions containing the desired product were combined, concentrated and co-evaporated with methanol (3 × 20 mL) to give **3g·TFA** (16 mg, 13%) as a pale yellow oil ^1^H-NMR (600 MHz; CD_3_OD) δ 3.51 (2H, t, *J* = 6.6 Hz, C*H*_2_CH_2_N^+^), 5.05 (2H, t, *J* = 6.6 Hz, C*H*_2_N^+^), 6.66–6.70 (2H, m, 4′′-H, 6′′-H), 7.37 (1H, d, *J* = 8.3 Hz, 3′′-H), 7.57–7.62 (6H, m, Ph), 7.74–7.77 (4H, m, Ph), 8.95 (2H, d, *J* = 1.6 Hz, 2-H, 6-H), 9.02 (1H, t, *J* = 1.6 Hz, 4-H); ^13^C-NMR (125 MHz; CD_3_OD) δ 38.0, 63.3 114.2, 117.9, 119.3, 128.7, 130.8, 131.6, 134.7, 135.0, 137.4, 142.0, 142.2, 142.9, 159.1; *m*/*z* [HRMS EI+] found [M − TFA]^+^ 430.0815. C_25_H_21_^79^BrNO^+^ requires 430.0807.

*1-(3-Hydroxybenzyl)-3,5-diphenylpyridinium***·***TFA* (**3h·TFA**). Compound **3h** was prepared according to procedure A, from 3-aminomethylphenol (62 mg, 0.50 mmol) and phenylacetaldehyde (300 mg, 2.50 mmol). The crude product was purified by preparative HPLC (retention time 14.4 min) to give **3h·TFA** as a pale yellow oil (147 mg, 65%). ν_max_ (neat)/cm^−1^ 3070, 2927, 1661, 1586, 1482; ^1^H- NMR (500 MHz; CD_3_OD) δ 5.88 (2H, s, C*H*_2_N^+^), 6.88 (1H, d, *J* = 7.9 Hz, 6′′-H), 6.97 (1H, s, 2′′-H), 7.02 (1H, d, *J* = 7.9 Hz, 4′′-H), 7.27 (1H, t, *J* = 7.9 Hz, 5′′-H), 7.57–7.63 (6H, m, Ph), 7.84–7.88 (4H, m, Ph), 9.04 (1H, s, 4-H), 9.30 (2H, s, 2-H, 6-H); ^13^C-NMR (125 MHz; CD_3_OD) δ 66.0, 116.4, 117.9, 120.5, 128.8, 130.8, 131.6, 131.9, 134.7, 136.1, 141.9, 142.3, 143.3, 159.7; *m*/*z* [HRMS ES+] found [M − TFA]^+^ 338.1513. C_24_H_20_NO^+^ requires 338.1539.

*1-(2,3-Dihydroxypropyl)-3,5-diphenylpyridinium***·***TFA* (**3i·TFA**). Compound **3i** was prepared according to procedure A, from 3-aminopropane-1,2-diol (45 mg, 0.49 mmol) and phenylacetaldehyde (300 mg, 2.50 mmol). The crude product was purified by preparative HPLC (retention time 13.0 min) to give **3i·TFA** as a pale yellow oil (147 mg, 72%). ν_max_ (neat)/cm^−1^ 3292, 3070, 1667, 1597, 1485; ^1^H-NMR (500 MHz; CD_3_OD) δ 3.60 (1H, dd, *J* = 11.4 and 6.0 Hz, C*H*HOH), 3.76 (1H, dd, *J* = 11.4 and 4.7 Hz, CH*H*OH), 4.15–4.18 (1H, m, C*H*OH), 4.74 (1H, dd, *J* = 13.2 and 8.5 Hz, C*H*HN^+^), 4.97 (1H, dd, *J* = 13.2 and 3.0 Hz, CH*H*N^+^), 7.57–7.64 (6H, m, Ph), 7.87–7.91 (4H, m, Ph), 9.03 (1H, t, *J* = 1.7 Hz, 4-H), 9.21 (2H, d, *J* = 1.7 Hz, 2-H, 6-H); ^13^C-NMR (125 MHz; CD_3_OD) δ 64.3, 65.7, 71.9, 128.8, 130.8, 131.5, 134.9, 141.8, 142.5, 142.9; *m*/*z* [HRMS EI] found [M − TFA]^+^ 306.1488. C_20_H_20_NO_2_^+^ requires 306.1489.

*1-(6-Hydroxyhexyl)-3,5-diphenylpyridinium***·***TFA* (**3j·TFA**). Compound **3j** was prepared according to procedure A, from 6-hydroxyhexylamine (59 mg, 0.50 mmol) and phenylacetaldehyde (300 mg, 2.50 mmol). The crude product was purified by preparative HPLC (retention time 15.3 min) to give **3j·TFA** as a pale yellow oil (153 mg, 69%). ν_max_ (neat)/cm^−1^ 3070, 2932, 1598, 1484; ^1^H-NMR (500 MHz; CD_3_OD) δ 1.49–1.85 (6H, m, 3 × C*H*_2_), 2.14–2.19 (2H, m, C*H*_2_), 3.67 (2H, t, *J* = 6.3 Hz, C*H*_2_OH), 4.75 (2H, t, *J* = 7.8 Hz, C*H*_2_N^+^), 7.58–7.65 (6H, m, Ph), 7.88–7.92 (4H, m, Ph), 9.03 (1H, t, *J* = 1.6 Hz, 4-H), 9.29 (2H, d, *J* = 1.6 Hz, 2-H, 6-H); ^13^C-NMR (125 MHz; CD_3_OD) δ 26.2, 26.8, 28.9, 32.5, 62.6, 63.4, 128.8, 130.8, 131.6, 134.9, 141.8, 142.0, 143.2; *m*/*z* [HRMS ES+] found [M − TFA]^+^ 332.2018. C_23_H_26_NO^+^ requires 332.2014.

*1-(4-Carboxybutyl)-3,5-diphenylpyridinium***·***TFA* (**3k·TFA**). Compound **3k** was prepared according to procedure A, from 4-aminobutyric acid (51 mg, 0.49 mmol) and phenylacetaldehyde (300 mg, 2.50 mmol). The crude product was purified by preparative HPLC (retention time 14.2 min) to give **3k·TFA** as a pale yellow oil (134 mg, 63%). ν_max_ (neat)/cm^−1^ 3200, 2943, 1668, 1598, 1483; ^1^H-NMR (500 MHz; CD_3_OD) δ 2.43 (2H, m, C*H*_2_CH_2_CO_2_H), 2.56 (2H, t, *J* = 6.8 Hz, C*H*_2_CO_2_H), 4.82 (2H, t, *J* = 7.3 Hz, C*H*_2_N^+^), 7.56–7.64 (6H, m, Ph), 7.88–7.92 (4H, m, Ph), 9.00 (1H, t, *J* = 1.6 Hz, 4-H), 9.28 (2H, d, *J* = 1.6 Hz, 2-H, 6-H); ^13^C-NMR (125 MHz; CD_3_OD) δ 27.6, 31.3, 62.7, 128.8, 130.8, 131.5, 134.9, 141.9, 142.2, 143.1, 175.7; *m*/*z* [HRMS ES+] found [M − TFA]^+^ 318.1485. C_21_H_20_NO_2_^+^ requires 318.1494.

*1-Cyclohexyl-3,5-diphenylpyridinium***·***TFA* (**3l·TFA**). Compound **3l** was prepared according to procedure A from cyclohexylamine (50 mg, 0.50 mmol) and phenylacetaldehyde (300 mg, 2.50 mmol). The crude product was purified by preparative HPLC (retention time 16.2 min) to give **3l·TFA** as a pale yellow oil (113 mg, 53%). ^1^H-NMR (500 MHz; CD_3_OD) δ 1.46 (1H, app. qt, *J* = 13.2 and 3.6 Hz, 4′′-H_ax_), 1.63 (2H, app. q, *J* = 10.2 Hz, 2 × 2′′-H_ax_), 1.82 (1H, br d, *J* = 13.2 Hz, 4′′-H_eq_), 2.05 (2H, br d, *J* = 13.8 Hz, 2 × 2′′-H_eq_), 2.18 (2H, qd, *J* = 12.3 and 3.6 Hz, 2 × 3′′-H_ax_), 2.32 (2H, br d, *J* = 10.6 Hz, 2 × 3′′-H_eq_), 4.82 (1H, m, C*H*N^+^), 7.56–7.64 (6H, m, Ph), 7.89–7.92 (4H, m, Ph), 9.00 (1H, d, *J* = 1.6 Hz, 4-H), 9.25 (2H, d, *J* = 1.6 Hz, 2-H, 6-H); ^13^C-NMR (125 MHz; CD_3_OD) δ 25.5, 26.6, 34.3, 74.3, 129.0, 130.8, 131.5, 135.0, 140.5, 142.1, 143.3; *m*/*z* (ES+) 314 (M^+^, 100%), 232 (12); *m*/*z* [HRMS ES+] found [M − TFA]^+^ 314.1906. C_23_H_24_N^+^ requires 314.1909.

*3,5-Bis-(4-hydroxyphenyl)-1-(4-hydroxyphenethyl)pyridinium***·***TFA* (**3o·TFA**). Compound **3o** was prepared according to procedure A, from tyramine**·**HCl (5.0 mg, 0.028 mmol) and 4-hydroxy-phenylacetaldehyde [22] (17 mg, 0.13 mmol). The crude product was purified by preparative HPLC (retention time 13.5 min) to give **3o·TFA** as a pale yellow oil (9.2 mg, 66%). ^1^H-NMR (600 MHz; CD_3_OD) δ 3.28 (2H, t, *J* = 6.7 Hz, C*H*_2_CH_2_N^+^), 4.87 (2H, t, *J* = 6.7 Hz, C*H*_2_N^+^), 6.73 (2H, d, *J* = 8.2 Hz, 3′′-H, 5′′-H), 6.94–6.98 (6H, m, 2 × (3′H, 5′-H), 2′′-H, 6′′-H), 7.57 (d, *J* = 8.2 Hz, 2 × 2′H, 6′-H), 8.71 (2H, d, *J* = 1.6 Hz, 2-H, 6-H), 8.77 (1H, t, *J* = 1.6 Hz, 4-H); ^13^C-NMR (150 MHz; CD_3_OD) δ 37.7, 64.6, 116.9, 117.5, 125.6, 127.5, 130.1, 131.3, 139.2, 139.9, 142.5, 158.3, 161.2; *m*/*z* [HRMS ES+] found [M − TFA]^+^ 384.1602. C_25_H_22_NO_3_^+^ requires 384.1600.

*1-(4-Hydroxyphenethyl)-3,5-di(thiophen-2-yl)pyridinium***·***TFA* (**3p·TFA**). Compound **2p** was prepared according to procedure A, from tyramine**·**HCl (5.0 mg, 0.028 mmol) and thiophen-2-yl-acetaldehyde [22] (17 mg, 0.14 mmol). The crude product was purified by preparative HPLC (retention time 13.5 min) to give **3p·TFA** as a pale yellow oil (5.6 mg, 42%). ^1^H-NMR (600 MHz; CD_3_OD) δ 3.29 (2H, t, *J* = 6.8 Hz, C*H*_2_CH_2_N^+^), 4.87 (2H, t, *J* = 6.8 Hz, C*H*_2_N^+^), 6.73 (2H, d, *J* = 8.5 Hz, 3′′-H, 5′′-H), 6.99 (2H, d, *J* = 8.5 Hz, 2′′-H, 6′′-H), 7.27 (2H, dd, *J* = 4.9 and 3.8 Hz, 4′-H), 7.75–7.78 (4H, m, 2 × 3′′-H, 5′′-H), 8.79 (1H, t, *J* = 1.7 Hz, 4-H), 8.83 (2H, d, *J* = 1.7 Hz, 2-H, 6-H); ^13^C-NMR (150 MHz; CD_3_OD) δ 37.5, 64.8, 116.9, 127.3, 129.7, 130.3, 131.2, 131.3, 136.4, 136.7, 136.9, 139.7, 158.3; *m*/*z* [HRMS ES+] found [M − TFA]^+^ 364.0820. C_21_H_18_NOS_2_^+^ requires 364.0830.

*1-(4-Hydroxyphenethyl)-3,5-bis-(4-methoxyphenyl)pyridinium***·***TFA* (**3q·TFA**). Compound **3q** was prepared according to procedure A, from tyramine**·**HCl (10 mg, 0.056 mmol) and 4-methoxy-phenylacetaldehyde [24] (38 mg, 0.25 mmol). The crude product was purified by preparative HPLC (retention time 16.2 min) to give **3q·TFA** as a pale yellow oil (17 mg, 58%). ^1^H-NMR (600 MHz; CD_3_OD) δ 3.29 (2H, t, *J* = 6.7 Hz, C*H*_2_CH_2_N^+^), 3.88 (6H, s, 2 × OMe), 4.90 (2H, t, *J* = 6.7 Hz, C*H*_2_N^+^), 6.74 (2H, d, *J* = 8.5 Hz, 3′′-H, 5′′-H), 6.97 (2H, d, *J* = 8.5 Hz, 2′′-H, 6′′-H), 7.11 (4H, d, *J* = 8.7 Hz, 3′-H, 5′-H), 7.68 (4H, d, *J* = 8.7 Hz, 2′-H, 6′-H), 8.78 (2H, d, *J* = 1.6 Hz, 2-H, 6-H), 8.83 (1H, t, *J* = 1.6 Hz, 4-H); ^13^C- NMR (150 MHz; CD_3_OD) δ 37.7, 56.0, 64.7, 116.1, 116.9, 126.9, 127.5, 130.3, 131.3, 139.8, 140.4, 142.3, 158.3, 163.1; *m*/*z* [HRMS ES+] found [M − TFA]^+^ 412.1909. C_27_H_26_NO_3_^+^ requires 412.1913.

*1-(4-Hydroxyphenethyl)-3,5-di(isopropyl)pyridinium***·***TFA* and *1-(4-Hydroxyphenethyl)-2-isobutyl-3,5-di(isopropyl)pyridinium***·***TFA* (**3r·TFA** and **1r·TFA**). Compounds **3r** and **1r** were prepared according to procedure **A**, from tyramine HCl (52 mg, 0.30 mmol) and isovaleraldehyde (120 mg, 1.4 mmol). The crude products were purified by preparative HPLC (retention times 15.3 min (**3r**) and 16.2 min (**1r**)) to give **3r·TFA** (6 mg, 5%) and **1r·TFA** (13 mg, 10%) as pale yellow oils. Compound **3r**: ^1^H-NMR (500 MHz; CD_3_OD) δ 1.28 (12H, d, *J* = 6.7 Hz, 2 × CH(C*H*_3_)_2_), 3.05–3.17 (4H, m, C*H*(CH_3_)_2_, C*H*_2_CH_2_N^+^), 4.77 (2H, t, *J* = 6.6 Hz, C*H*_2_N^+^), 6.65 (2H, d, *J* = 8.4 Hz, 3′-H, 5′-H), 6.79 (2H, d, *J* = 8.4 Hz, 2′-H, 6′-H), 8.29 (2H, s, 2-H, 6-H), 8.34 (1H, s, 4-H); ^13^C-NMR (125 MHz; CD_3_OD) δ 23.2, 33.2, 37.6, 64.1, 116.8, 127.2, 130.9, 141.7, 143.2, 150.7, 158.2; *m*/*z* [HRMS ES+] found [M − TFA]^+^ 284.2019. C_19_H_26_NO^+^ requires 284.2014. Compound **1r**: ^1^H-NMR (500 MHz; CD_3_OD) δ 1.12 (6H, d, *J* = 6.7 Hz, CH(C*H*_3_)_2_), 1.20 (6H, d, *J* = 6.9 Hz, CH(C*H*_3_)_2_), 1.31 (6H, d, *J* = 6.8 Hz, CH(C*H*_3_)_2_), 1.97–2.03 (1H, m, C*H*(CH_3_)_2_), 2.92 (2H, d, *J* = 7.5 Hz, 2 × *CH*_2_CH(CH_3_)_2_), 2.98 (1H, hept, *J* = 6.9 Hz, C*H*(CH_3_)_2_), 3.15 (2H, t, *J* = 6.4 Hz, C*H*_2_CH_2_N^+^), 3.32–3.36 (1H, m, C*H*(CH_3_)_2_), 4.86 (2H, t, *J* = 6.4 Hz, C*H*_2_N^+^), 6.65 (2H, d, *J* = 8.5 Hz, 3′-H, 5′-H), 6.74 (2H, d, *J* = 8.5 Hz, 2′-H, 6′-H), 8.14 (1H, s, 6-H), 8.30 (1H, s, 4-H); ^13^C-NMR (125 MHz; CD_3_OD) δ 22.2, 23.0, 23.5, 31.0, 31.5, 32.8, 36.8, 37.0, 61.7, 116.8, 127.1, 131.1, 142.7, 143.3, 147.7, 150.6, 153.7, 158.3; *m*/*z* [HRMS ES+] found [M − TFA]^+^ 340.2650. C_23_H_34_NO^+^ requires 340.2640.

## 5. Conclusions

New reaction conditions have been developed that promote the condensation of arylacetaldehydes with a variety of amines to yield 1,3,5-pyridinium salts **3**. The reaction is promoted by phosphate and can be carried out under conditions suitable for intrinsically unstable substrates such as arylaldehydes: the reaction is derived from the Chichibabin pyridine synthesis. These results could underpin the involvement of phosphate-containing molecules (e.g., inorganic phosphate, DNA, ATP) as catalysts for the production of many alkaloids in vivo. The evaluation of a selection of 1,3,5-trisubstituted pyridinium salts as bacteriocides prompted the discovery of novel *S. aureus* inhibitors in the low μg/mL range. These preliminary results may pave the way towards the discovery of new antibiotics based on the yet unexploited 1,3,5-trisubstituted pyridinium scaffold.

## Figures and Tables

**Table 1:** Influence of buffer conditions on the formation of **3a** (Scheme 2ii) ^1^.

Entry	Buffer	Yield of 3a
1	KH_2_PO_4_ (KPi)	48%
2	Glc-1-P	40%
3	PPi	23%
4	UMP	53%
5	B(OH)_3_	<1%
6	HEPES	3%
7	Tris	<1%
8	water only	<1%

^1^ *Reaction conditions*: **4a** (5 equiv.), **6a** (1 equiv.), 0.1 M buffer at pH 6, 60 °C, 12 h. Yields of **3a** were determined by HPLC analysis.

**Table 2:**
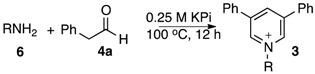
One-step phosphate mediated synthesis of 1,3,5-pyridiniums **3** ^1^.

Amine	R	Product	Isolated Yield	Amine	R	Product	Isolated Yield
**6a**	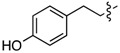	**3a**	70%	**6h**	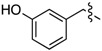	**3h**	65%
**6b**	PhCH_2_CH_2_	**3b**	54%	**6i**	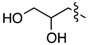	**3i**	72%
**6c**	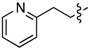	**3c**	52%	**6j**	(CH_2_)_6_OH	**3j**	69%
**6d**	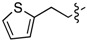	**3d**	38%	**6k**	(CH_2_)_3_CO_2_H	**3k**	63%
**6e**	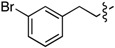	**3e**	50%	**6l**	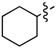	**3l**	53%
**6f**	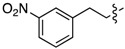	**3f**	45%	**6m**	Ph	**3m**	0%
**6g**	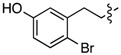	**3g**	13% ^2^	**6n**	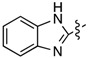	**3n**	0%

^1^ *Reaction conditions:* **4a** (5 equiv.), amine **6** (1 equiv.), 0.25 M KPi buffer:MeOH (1:1) at pH 6, 100 °C, 12 h; ^2^ **4a** (1.2 equiv.), amine **6** (1 equiv.), 0.1 M KPi buffer at pH 6, 60 °C, 12 h (Pictet-Spengler reaction conditions).

**Table 3:**
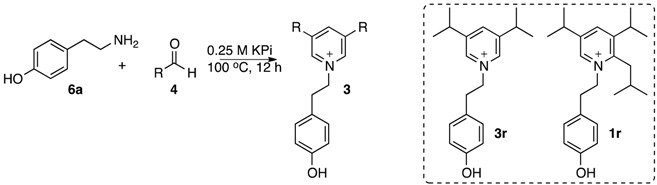
Versatility of the phosphate-mediated pyridinium synthesis towards aldehydes **4** ^1^.

Aldehyde	R	Product	Yield
**4a**	PhCH_2_	**3a**	70%
**4o**	4-HOC_6_H_4_CH_2_	**3o**	66%
**4p**	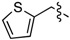	**3p**	42%
**4q**	4-MeOC_6_H_4_CH_2_	**3q**	58%
**4r**	CH(CH_3_)_2_	**3r (1r)** ^2^	5(10)%
**4s**	C_2_H_5_	**3s**	0%

^1^ Reaction conditions: **4** (5 equiv.), amine **6a** (1 equiv.), 0.25 M KPi buffer:MeOH (1:1) at pH 6, 100 °C, 12 h; ^2^ Reaction also yielded **1r**.

**Table 4:** MIC data for a range of the pyridiniums **3** determined from MIC tests.

Compound	^1^ MIC (μg/mL)	Compound	^1^ MIC (μg/mL)
**3a**	n.d.	**3h**	64
**3b**	32	**3i**	n.d.
**3c**	n.d.	**3j**	64
**3d**	64	**3k**	n.d.
**3e**	16	**3o**	32
**3f**	64	**3q**	32
**3g**	16	**3r**	n.d.

^1^ No growth inhibition detected (n.d.) represents compounds with MIC > 256 μg/mL. MIC for kanamycin against *S. aureus* has been reported as 3.5 μg/mL [28].
